# The emerging role of alternatively activated macrophages to treat acute liver injury

**DOI:** 10.1007/s00204-024-03892-2

**Published:** 2024-11-06

**Authors:** Chris Humphries, Melisande L. Addison, James W. Dear, Stuart J. Forbes

**Affiliations:** 1 Centre for Cardiovascular Science, Queen’s Medical Research Institute, University of Edinburgh, 47 Little France Drive, Edinburgh, UK; 2 Centre for Regenerative Medicine, Institute for Regeneration and Repair, University of Edinburgh, 4-5 Little France Drive, Edinburgh, EH16 4UU UK

**Keywords:** Cell therapy, Macrophages, Liver injury, Liver regeneration, Inflammation, Acetaminophen overdose

## Abstract

Acute liver injury (ALI) has a clear requirement for novel therapies. One emerging option is the use of alternatively activated macrophages (AAMs); a distinct subtype of macrophage with a role in liver injury control and repair. In this comprehensive review, we provide an overview of the current limited options for ALI, and the potential advantages offered by AAMs. We describe the evidence supporting their use from in vitro studies, pre-clinical animal studies, and human clinical trials. We suggest why the first evidence for the clinical use of AAMs is likely to be found in acetaminophen toxicity, and discuss the specific evidence for AAM use in this population, as well as potential applications for AAMs in other patient populations. The key domains by which the performance of AAMs for the treatment of ALI will be assessed are identified, and remaining challenges to the successful delivery of AAMs to clinic are explored.

## Introduction

Acute liver injury (ALI) can come from a variety of causes including toxins, drug overdose such as acetaminophen (APAP), viruses and autoimmune diseases and can present with deranged liver function tests and coagulopathy. Whatever the aetiology, this can result in hepatic necrosis, liver inflammation and impaired immunity. If unresolved, ALI can progress to acute liver failure (ALF) which has a high mortality rate. Once ALI is established, there are no specific therapies apart from supportive care, emphasising that novel therapies are required.

In this review, we will discuss how macrophage therapy has potential for the treatment of ALI. We explain how liver macrophages respond to ALI and have a particular focus on alternatively activated macrophages (AAMs), which are a phenotypically distinct subtype of monocyte-derived macrophage, that play a significant role in liver injury control and repair processes (Ramachandran et al. 2012). The evidence from lab bench to human studies is explored, and ongoing clinical trials are identified. We speculate on the future applications of AAMs, and identify key challenges which translational researchers face in their attempts to bring these cells to clinic.

Briefly, in contrast to pro-inflammatory classically activated macrophages (CAMs, also described as subtype M1), AAMs are typically considered to be ‘anti-inflammatory’ (M2). There have been attempts to sub-classify M2 macrophage in vitro (M2a–M2d) by their production pathways and cell markers, but cell polarisation is rarely terminal or homogenous, and differences can be seen when products studied in vitro are administered in vivo (Gharavi et al. 2022). Variation in production processes between laboratories may lead to differences in gene expression even for two cell populations given the same broad label, and cell marker expression may change across disease stages, making generalisations across the literature challenging (Murray et al. 2014).

The AAM subtype is considered to exhibit anti-inflammatory and wound healing properties and has been termed ‘M2a’ by some. Due to common membrane markers across cell types, and challenges classifying in vivo behaviour, we hereafter only refer to ‘AAMs’ in this review to describe M2 cells which are produced by stimulating monocytes with IL-4 and IL-13 in vitro, and have evidence to support their use in ALI.

Cytotherapy with AAMs offers a novel approach in comparison with traditional small molecule, antibody, or peptide therapeutics. Rather than targeting specific receptors or metabolic pathways, AAMs aim to provide an increased quantity of the cellular machinery required to alter the pathophysiological processes of the recipient. This delivers exciting potential for a ground-breaking new technology. However, clinical research facilities will face novel challenges regarding production pathways, trial staff expertise, and the development of methodology for demonstrating study product efficacy, pharmacokinetics and pharmacodynamics.

## Novel treatments are needed to prevent ALF

It is important to recognise that all patients with ALF can be considered to have experienced ALI as a primary cause of their condition, prior to developing hepatic encephalopathy. The care of ALF is largely supportive, and although mortality rates have improved over decades, the only definitive treatment for patients who do not recover from ALF with supportive care is orthotopic liver transplantation (OLT) (Reuben et al. 1998). OLT requires lifelong immunosuppression, has associated morbidity and mortality, and is limited by donor liver availability.

There are very few cause-specific treatments with any evidence to support claims that their use in ALI may prevent the development of ALF or reduce the requirement for OLT (Stravitz and Lee 2019). Even acetylcysteine, used for decades as an antidote following APAP ingestion, is only effective at preventing the development of ALI following APAP ingestion; it does not convincingly reverse established injury (Lee et al. 2009). If a new treatment could be developed which prevents the progression of ALI to ALF, it would be life-saving, as well as reducing the requirement for OLT.

As discussed below, AAMs have been shown to be effective in a mouse model of ALI, transitioning the initial inflammatory environment of ALI to one characterised by features of resolution and repair. They act to phagocytose necrotic tissue, reduce pro-inflammatory cytokine release, and stimulate hepatocyte proliferation (Starkey Lewis et al. 2020). If AAMs are effective in humans with ALI, then clinicians would have a new therapeutic option to support the care of this patient group, filling a currently unmet need. This may also provide future scope for testing AAMs in other pathologies characterised by necrosis and inflammation.

## Who has ALI? Defining and identifying a population for research

Pre-clinical identification of patient populations that have potential to benefit from AAMs is a challenge. Defining which patients have ALI is not straightforward; recognition criteria for ALI in the absence of ALF are inconsistently defined across clinical settings. For example, it has been suggested that ‘ALF criteria without encephalopathy’ may be used clinically, whilst European Association for the Study of the Liver clinical guidelines recommend different classification criteria which vary according to the mechanism of drug-induced liver injury (DILI) (Koch et al. 2017; European Association for the Study of the Liver 2019). In the context of early-phase clinical trials, it has been suggested that small rises in alanine aminotransferase (ALT > 3 × upper limit of normal) should lead clinicians to suspect DILI (European Association for the Study of the Liver 2019).

Whilst the precise criteria used to define ALI may vary, the data regarding causes at a population level are relatively consistent when threshold ALT values are used to define the population by searching serology results, and suggest that DILI (predominantly APAP), ischaemic hepatitis, biliary obstruction and viral hepatitis account for the majority of ALI (Galvin et al. 2015; Con et al. 2019). Notably, ischaemic hepatitis and biliary obstruction have obvious interventions with a good evidence base (the restoration of perfusion, or surgical management respectively), and viral hepatitis predominates in developing countries rather than high-income countries (Patterson et al. 2020).

Predicting which patients with ALI will develop ALF at an individual level lacks sufficiently robust tools to allow for personalised care (European Association for the Study of the Liver et al. 2017). At a population level, APAP toxicity is the most prevalent cause of admission and mortality in the United Kingdom, Europe, and the USA, whilst viral hepatitis is the leading cause in many developing countries (Koch et al. 2017; Patterson et al. 2020; Polson and Lee 2005; Office for National Statistics 2023). Although the mortality rate associated with ALF from viral hepatitis is estimated at 50%, the implementation of immunisation and effective antivirals is likely to be the most clinically impactful action, and whether additional therapy with AAMs would help in the context of viral disease is debatable (Li et al. 2020).

As translational research in regenerative medicine is predominantly undertaken in high-income countries, and there is a clear rationale for the therapeutic action of AAMs in DILI, the target population for the use of AAMs in acute scenarios has focussed on DILI, with APAP toxicity representing a robustly researched and clearly defined DILI population in whom the role of macrophages has been well characterised in mice. Mouse models of APAP toxicity are well established, providing an excellent test bed for pre-clinical studies of novel advanced therapies, and there are clinical research centres with significant experience in recruiting this cohort of patients to clinical trials (Starkey Lewis et al. 2020; Jaeschke et al. 2021; Thanacoody et al. 2013; Morrison et al. 2019).

When compared to other mechanisms of DILI (such as idiosyncratic DILI), patients who suffer APAP DILI typically have a clear diagnosis at an early stage, a predictable time course to their injury, and a well-defined pattern of biomarker release with alanine aminotransferase. These factors all lend themselves to the recruitment and study of clinical trial participants.

For all these reasons, it is reasonable to assume that the first patient population in which the use of AAMs in ALI is studied will be APAP toxicity. If AAMs are found to be a viable product in this setting, the lack of a coherent ALI definition may mean that any threshold treatment or biomarker values used in the clinical trials become de facto standards to identify patients who should receive them as treatment.

## The natural history of macrophage populations in APAP ALI

APAP toxicity produces a characteristic pattern of centrilobular necrosis (around the central vein, acinar zone 3), and in more severe cases, this can extend, causing massive necrosis (Krishna 2017). The centrilobular necrosis pattern occurs because hepatocytes in this region have a greater concentration of cytochrome P450 enzymes, leading to increased APAP metabolism in this region, and consequently, increased production of its toxic by-product: *N*-acetyl-*p*-benzoquinone imine (NAPQI) (Butler et al. 2018). NAPQI is detoxified by glutathione, but when glutathione supplies become exhausted, NAPQI causes mitochondrial protein adduct formation, leading to c-jun N-terminal kinase (JNK) activation, which subsequently impacts mitochondrial respiration (Jaeschke et al. 2021). The consequent cascade results in the release of endonucleases, DNA fragmentation and necrotic cell death (Bajt et al. 2006; Jaeschke and Ramachandran 2020).

In mouse models of APAP ALI, the hepatic macrophage population increases over approximately 72 h, despite Kupffer cell (KC) numbers falling initially (KCs are located at the liver sinusoid endothelium, receiving blood supplied by the portal vein, and consequently are involved early in liver injury) (Ju et al. 2002). The rapid increase in hepatic macrophage numbers is primarily driven by infiltrating circulating monocytes, which differentiate to form macrophages (Zigmond et al. 2014). For completeness, we also note that there is a population of peritoneal macrophages which may be recruited to subcapsular liver injuries, but have not been found to play any significant role in DILI (Wen et al. 2021).

Early work suggested that in ALI, circulating monocytes differentiate to ‘pro-inflammatory’ CAMs, and proliferation of the KC pool produces macrophages with phenotypic similarities to ‘anti-inflammatory’ AAMs (Antoniades et al. 2012). However, further research does not support macrophage ontogeny being so simple in the ALI landscape. It now appears that when monocytes infiltrate the liver in response to ALI, the polarisation is initially inflammatory, and then monocytes begin polarising to a pro-resolution phenotype before KC numbers have recovered (Zigmond et al. 2014).

The interaction between infiltrating monocyte and KC populations is conflicting: monocytes have been shown by some to be capable of restoring KC populations, and can rapidly acquire markers of KC function, whilst other data suggest that they play no role in recovery of KC populations after APAP ALI (Scott et al. 2016; Bonnardel et al. 2019). In addition, it is clear that liver macrophage phenotypes do not fit neatly into binary pro- or anti-inflammatory classifications: for example, it was recently shown that KCs drive hepatocyte proliferation by secreting IL-6 (a cytokine classically considered pro-inflammatory) (Li et al. 2023). Whilst the phenotypes, interactions, functionality and translational potential of liver macrophages are increasingly well described, it is not always clear when findings in alternative models of injury can be applied to ALI (Guillot and Tacke 2024).

Study of murine macrophage behaviours in APAP ALI suggests that initial inflammation from APAP toxicity triggers the release of pro-inflammatory cell signalling molecules by KCs (e.g. IL-1α), resulting in chemoattraction of neutrophils and Ly6C^hi^ monocytes into the niche, which further amplifies the level of inflammation, and contributes to the injury (Zhang et al. 2018). In the early stages of injury, pro-inflammatory cells predominate, as AAM polarisation takes significantly longer to occur (Derlindati et al. 2015). By 72 h after injury, monocytes become Ly6c^lo^, associated with a pro-restorative phenotype characterised by high levels of phagocytosis, promotion of hepatocyte proliferation and reduced inflammatory cytokine release (Antoniades et al. 2012; Graubardt et al. 2017). These concepts are the subject of debate, and whilst conflicting results may make the choice of cell product more challenging, the use of robust pre-clinical experimental controls and hard end-points (such as histological liver necrosis and cell proliferation) can support progression from pre-clinical studies to clinical trials (Starkey Lewis et al. 2020; Jaeschke 2018).

The plasticity of infiltrating macrophages in ALI fosters some uncertainty regarding the importance of dose timing to the success of AAM supplementation in APAP ALI. In humans, early monocytopenia has been identified as a poor prognostic factor in APAP overdose, which may reflect the influx of monocytes to the hepatic niche from the circulation, an increased pro-inflammatory response, and/or a depleted supply for the production of additional pro-restorative macrophages in later stages of injury (Moore et al. 2017). Mouse studies by Starkey-Lewis et al. demonstrated no improvement in survival for mice given AAMs at 4 h post-APAP injury; it was when AAMs were administered 16 h post-injury, and mice culled at 84 h that differences in both necrosis and biomarker response were identified. The failure of human AAMs to demonstrate effect in immunocompromised mice, whilst delivering a significant reduction in liver necrosis in immunocompetent mice, suggests that immune system cross-talk is critical to the mechanism of AAMs in treating ALI. This is observed in the increased number of Ly6c^lo^ macrophages present in the circulation of mice treated with AAMs (Starkey Lewis et al. 2020). Further studies would now be helpful to elucidate the precise mechanisms of AAMs in APAP ALI.

Clinical concerns regarding the safety and justifiability of liver biopsy in human APAP ALI mean it is likely to remain unclear how directly mouse models of disease can be extrapolated to the use of AAMs in humans. Evidence of efficacy in humans is likely to be primarily provided by biomarker interpretation (Humphries and Dear 2023). The potential impact of AAMs in humans is arguably far greater in humans than mice—the resolution of necrosis in mouse APAP ALI typically takes only 5 days, whilst in humans, the histological changes may not resolve for up to 4 months (Portmann et al. 1975). AAMs could conceivably, therefore, have a significantly longer window of opportunity in humans than seen in mouse models.

## The evidence supporting the efficacy of AAMs in ALI

### In vitro studies

**Fig. 1:**
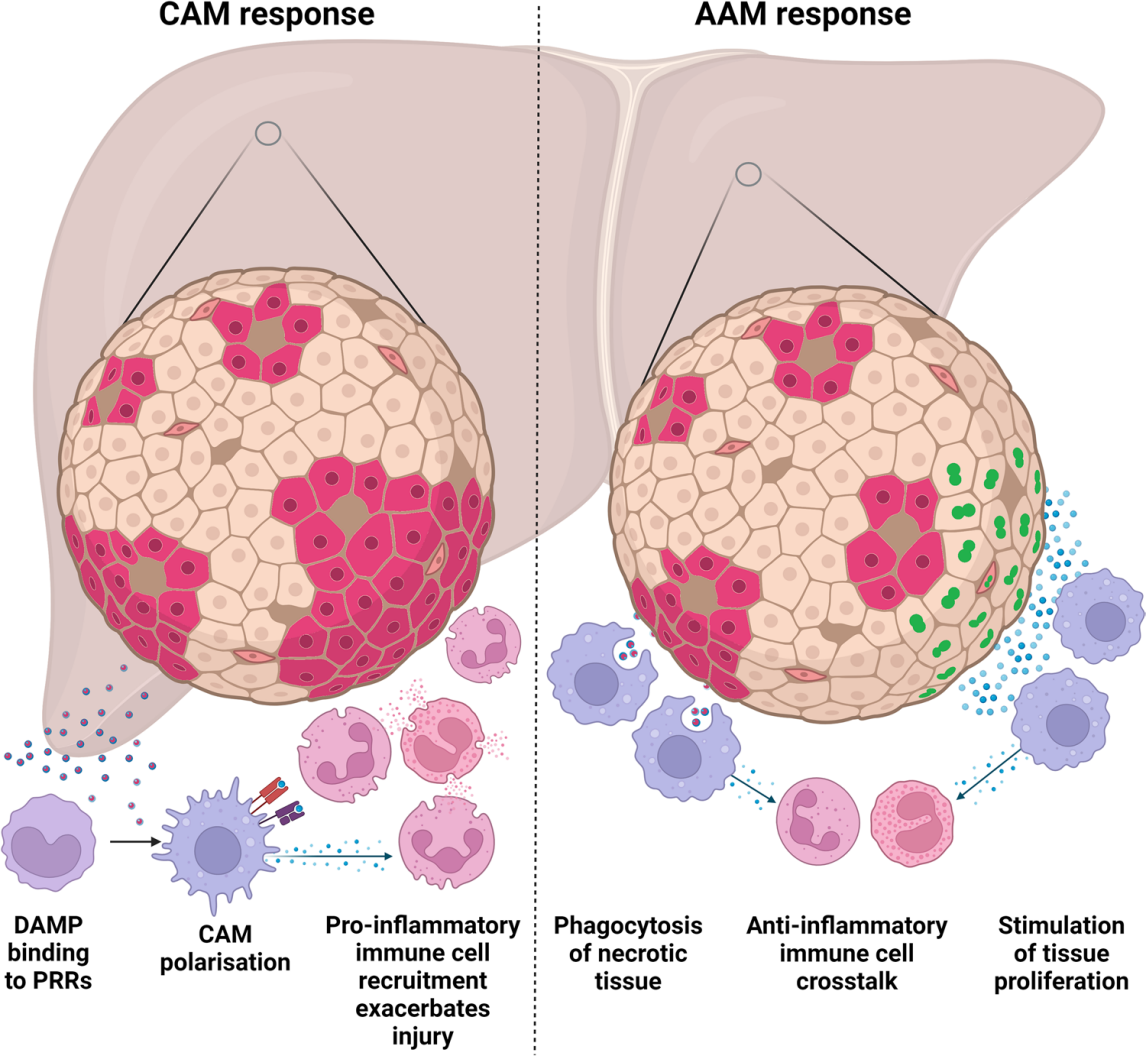
The mechanisms by which AAMs are thought to produce their effect in ALI. (1) Phagocytosis of necrotic tissue by AAMs; (2) promotion of hepatocyte and endothelial tissue proliferation; (3) the pro-inflammatory immune response is downregulated via AAM cytokine release. Image created with biorender.com

As the effects of AAMs are multiple—stimulating the proliferation of other cell types, phagocytosing necrotic debris, and reducing the inflammatory cytokine secretion of other cells—the ability of in vitro experiments to demonstrate efficacy is limited to demonstrating the principle of individual behaviours they are thought to exhibit in vivo. Figure 1 illustrates the mechanisms of action in ALI.

In vitro work has demonstrated little difference between AAMs produced using combined IL-4 and IL-13 treatment when compared to IL-4 or IL-13 alone, and relevant evidence resulting from both methods is, therefore, discussed here (Scott et al. 2023). Immortalised macrophage-like cell lineages, such as THP-1 and RAW264.7, have been shown to have significant differences in their phenotypes, and we, therefore, do not review work using cell lineages (Tarique et al. 2015).

Mouse AAMs have been shown to have higher phagocytic capacity in vitro when compared to monocytes and CAMs, and to maintain their Ly6C^lo^ polarisation during the process (Starkey Lewis et al. 2020). Although total numbers of AAMs do reduce with time, human AAMs have been shown to persist for up to 12 days in cytokine-deficient medium before reverting to their undifferentiated precursor (Tarique et al. 2015).

Proteomic signature comparisons between macrophage subtypes indicate the potential proliferative function of AAMs. The AAM phenotype has been shown to have higher levels of membrane proteins involved in angiogenesis and phagocytosis (Becker et al. 2012). RNA-Seq analysis has identified marker genes expressed at high levels in human AAMs (but not CAMs or PBMCs) which have potential roles in processing endocytosed molecules, promoting tissue proliferation, contraction of necrotic areas, and the resolution of inflammatory disease (Gurvich et al. 2020). Co-culture of mouse AAMs with primary mouse hepatocytes supports the theory that AAMs mediate hepatocyte proliferation by secretion of hepatocyte growth factor (Yang et al. 2019).

The role of AAMs in reducing the cytokine secretion of other cells is more challenging to explore due to the degree of cross-talk which exists between immune cells. Human AAMs have been found to release cytokines that are not released by CAMs or PBMCs; IL-13, CCL13, CCL14, CCL17, CCL18, and CCL23 (Tarique et al. 2015; Martinez et al. 2006) These markers are primarily associated with monocyte and regulatory T-cell recruitment.

### In vivo studies

Whilst the role of inflammation in the initial liver injury of APAP ALI remains a subject of debate, it has been suggested in mice that the potency of the initial inflammatory immune response in APAP ALI is potentially maladaptive, aggravating liver injury. Monocytes are recruited by CCL2/CCR2 signalling, and use of CCR2-/- knockout mice, or CCL2 inhibition, demonstrates reduced liver injury after APAP, suggesting that the initial inflammatory response is not a required step in recovery after APAP toxicity (Mossanen et al. 2016). We can, therefore, infer that any impact of AAMs in reducing the initial inflammatory response if administered early in injury (rather than simply promoting resolution) is unlikely to be harmful. Conversely, depletion of AAMs during the resolution phase of liver injury has been associated with increased levels of ALT and hepatic necrosis, and reduced hepatocyte proliferation, suggesting that supplementation with exogenous AAMs has the potential to be helpful (Yang et al. 2019).

The pharmacokinetic behaviour of AAMs in pre-clinical studies suggests that after peripheral vein injection, AAMs pass through the lungs and localise rapidly to the liver and spleen, and are found throughout the liver parenchyma, with some preference for necrotic areas. The phagocytic and pro-proliferative activity of mouse AAMs has been clearly demonstrated in vivo, with up to 99% of AAMs found to be engaged in phagocytosis, and hepatocyte and endothelial tissue proliferation increasing by 8.4-fold. Interestingly, human AAMs administered to immunocompetent mice were also able to demonstrate a statistically significant reduction in liver necrosis and increase in hepatocyte proliferation (Starkey Lewis et al. 2020).

Mouse bone marrow-derived macrophage transcriptomics shows that AAM gene expression only begins climbing significantly at 12–24 h, well after CAM gene expression has begun falling from its peak, suggesting that exogenous AAMs administered earlier in ALI may allow restorative processes to begin more quickly (Roy et al. 2015). This is supported by the work of Lewis et al., who found that AAMs in APAP ALI were effective at reducing necrosis when given as early as 6 h after injury (Starkey Lewis et al. 2020).

It should be noted that despite the compelling evidence from mouse models, there are some significant differences between mouse and human macrophage cell markers, signal molecule functions, and resident macrophage localization (Wen et al. 2021; Barreby and Aouadi 2022). There are some limitations to the mouse model of APAP ALI, though these are increasingly well understood and anticipated (Jaeschke et al. 2021; Beger et al. 2015). It is not certain that AAMs will find an identical therapeutic niche regarding inflammatory and resolution stages in humans, and consequently, there is a clear need for human clinical trials to truly discover the tolerability and therapeutic applications of AAMs.

### Clinical trials with AAMs

To date, there have been no findings reported from clinical trials using macrophages in the treatment of APAP ALI. The bulk of macrophage trials have been for cancer, and these have focussed on the generation of pro-inflammatory CAM-type macrophage populations (which confusingly, in early work are often referred to as ‘Autologous Activated Macrophages’), in an effort to overcome tumour immunotolerance. Fever and cytokine release syndromes have been the most commonly observed toxicities, though dose-limiting toxicity has not been observed even with this pro-inflammatory macrophage phenotype (Reiss et al. 2021; Hennemann et al. 1998; Eymard et al. 1996).

There are clinical trials of macrophage therapy which are ongoing or have completed. However, the methods of obtaining the cells vary, as do the manufacturing processes, cell product heterogeneity, treatment indications, routes of administration, and duration of follow-up (Na et al. 2023). Trial population heterogeneity can make identifying signals of efficacy challenging, and as monocyte-derived macrophages infiltration and function in the liver may differ significantly in injured states, it is not feasible for phase 1 trials to use healthy volunteers, as they would not represent a suitable pharmacokinetic or phamacodynamic model to establish safety and tolerability. Without careful trial design, the recruitment of unwell patients may leave trials vulnerable to selection bias.

Autologous monocyte-derived macrophages (CD14+ monocytes matured in vitro using CSF-1) have been given in phase 1 and phase 2 trials for liver cirrhosis. The phase 1 trial found no serious adverse events related to the product. The phase 2 trial (MATCH) of 50 patients has not yet been published, but the 1-year follow-up data have been presented in abstract form as being well-tolerated, and associated with reduced morbidity and mortality (https://resolution-tx.com/resolution-therapeutics-founders-present-clinical-proof-of-concept-for-macrophage-cell-therapy-in-end-stage-liver-disease-at-aasld/). Long-term follow-up data have shown a marked reduction in mortality and improvement in transplant-free survival (Brennan et al. 2023). The performance of an engineered autologous AAM product (RTX001), which could enhance the regenerative properties of AAMs, will be studied in clinical trials beginning in 2024 (https://www.bioindustry.org/news-listing/macrophage-cell-therapy-shows-promise-for-end-stage-liver-disease.html).

Autologous product trials have clearly established the safety and feasibility of AAMs delivered by peripheral venous infusion. This, combined with expertise developed from decades of blood transfusion and organ transplant, has opened the door for an allogeneic product to be developed. The Macrophages for Acute Injury of the Liver (MAIL) Trial is a phase 1 trial of patients presenting with APAP ALI, which began recruiting in 2023, and will explore the safety and tolerability of AAMs in this patient group, whilst also allowing biomarker sub-studies to explore efficacy (10.1186/ISRCTN12637839).

Allogeneic infusion of other macrophage cell products has been undertaken previously. PBMC-derived regulatory macrophages were administered to two patients, and whole-body single-photon emission computed tomography (SPECT) demonstrated accumulation in the liver, spleen and haematopoetic bone marrow, with no evidence of pulmonary perfusion deficits (Hutchinson et al. 2011).

No matter how well clinical trials are designed, there may be some long-term effects of AAMs which only become apparent in post-market surveillance. The example of donor-derived bone marrow transplants leading to long-term complications only identified 25 years after administration demonstrates the need for continued scrutiny (Kelkar et al. 2023). By carefully selecting the populations most likely to benefit from AAMs, and clearly defining the circumstances in which they should be administered, clinicians can maximise the results of treatment whilst minimising the risk.

## Potential applications for AAMs in ALI

Therapeutic AAMs produced for ALI can be produced from monocytes obtained either by donor leukapheresis (peripheral blood mononuclear cells, PBMCs) or bone marrow harvest (bone marrow-derived macrophages, BMDMs). Leukapheresis is less painful for patients, and technically easier to perform.

Unlike the use of autologous products for chronic conditions such as liver cirrhosis, there is insufficient time for a patient with ALI to donate their own cells for culture. In addition, they may have lower numbers of cells available at leukapheresis. Donor monocytes are isolated, multiplied, and polarised with IL-4 and IL-13 to produce the required phenotype, and can be delivered by peripheral venous infusion (Fraser et al. 2017; Thomas et al. 2011). These characteristics bring AAM administration within the scope of any clinical facility capable of delivering blood products, as the product can be produced in advance and frozen—potentially permitting “urgent use” in the emergency room setting.

APAP ALI remains the most obvious use case for a viable ‘off-the-shelf’ product. The current absence of any therapeutic to reverse established damage, and significant lifetime costs associated with OLT (estimated at circa. $2,000,000 for patients over 20 years in the United States) make the clinical and economic arguments clear (Habka et al. 2015) Whilst the current focus is on identifying dose-limiting toxicity in patients who have achieved a peak ALT > 1000 U/L, it is not currently clear where cell therapy will sit in the evolution of ALI (Craig et al. 2012).

Whilst the wider expansion of indications for AAM therapy is dependent on evidence of safety, tolerability and efficacy in APAP ALI, it is possible to speculate regarding other clinical scenarios which may see adoption of AAMs. The preference will always be that management is based on robust clinical trials, but there are some clinical scenarios which make the delivery of trials extremely challenging (Dear 2023; Niu et al. 2021). The management of idiosyncratic DILI remains a clinical conundrum, with the only evidence-based therapy being cessation of the offending agent. In contrast to intrinsic DILI, the mechanisms involved in idiosyncratic DILI are suspected to be primarily due to macrophage activation, rather than just increased by it (Shan and Ju 2020). Whilst the pattern of injury in idiosyncratic DILI varies, specific drugs tend to have typical patterns, which may make the use of AAMs offending-drug specific, or restricted to patients with evidence of hepatocellular injury. Histologically, the necroinflammatory pattern found in many idiosyncratic DILI presentations, and the association of increased liver necrosis with severe or fatal outcomes suggests a potential role for AAMs beyond intrinsic DILI (Kleiner et al. 2014).

All secondary care facilities may manage cases of DILI, and DILI occurs in up to 5% of patients in phase 1 trials. The inevitability of DILI presentations in hospitals (and the duty of care held by clinical research facilities to minimise the chance of harm to participants) makes a case for the stocking of AAMs either locally or held at a central location permitting rapid dissemination and usage, if AAMs are found to be clinically effective and cost-effective (Mondaca et al. 2020).

Similar necroinflammatory patterns as those found in some DILI cases can be seen in severe autoimmune hepatitis (AIH), and it is conceivable that AAMs could have a role alongside immunosuppression, given the cytokine mechanisms thought to be in play (Gasmi and Kleiner 2020; Sirbe et al. 2021). Distinguishing AIH from DILI is complex, and therefore a therapeutic which is effective for both disease processes is appealing (Suzuki et al. 2011).

## Remaining challenges for AAMs

### Pharmacokinetic and pharmacodynamic assessment

The properties of cell products in humans cannot be assessed in the same way as those of small molecules; compartment localisation is likely to be tissue specific and cells may not exhibit classical half-lives due to having natural lifespans, with plasticity also potentially limiting the duration of their therapeutic activity (Aijaz et al. 2019). Methods developed in animal models to analyse activity, potency and biodistribution may be too invasive (e.g., intravital microscopy), or carry too much risk to be achievable in human subjects (Conlon and Mavilio 2018). Approaches for assessing cell distribution have been developed, including radiolabelled cell SPECT and MRI macrophage imaging, but even these cannot necessarily demonstrate localisation and function at a tissue level (Rodell et al. 2019).

The lack of information about tissue localisation in humans, and therefore whether there might be differential success in different models of disease, may make the selection of other candidate diseases difficult. For example, an understanding if AAMs demonstrate a pattern of distribution suggesting they are better suited to centrilobular or piecemeal necrosis may impact whether use is recommended in all causes of DILI.

In the absence of biopsy tissue (which is particularly unlikely to be obtained in APAP ALI, due to concerns around coagulopathy), biomarker studies are required to demonstrate impact (Humphries and Dear 2023). Whilst this could be understood as the development of new biomarkers, or quantifying and comparing levels of biomarkers between patient groups, this may also require re-examining what additional value can be extracted using existing biomarkers. Patients in AAM trials are unlikely to have good control groups for comparison, and so a robust understanding of the natural history of biomarker behaviour in the patient group, or techniques to allow prediction of biomarker behaviour are vital for mechanistic and dose-finding work.

Natural history studies are particularly important given that the importance of many damage-associated molecular pattern biomarkers and immune-signalling molecules in macrophage function is only established histologically, with no understanding of whether significant release into the circulation occurs. To understand the impacts of AAMs across the time course of disease states, there must be at least a qualitative basis for interpretation.

**Fig. 2:**
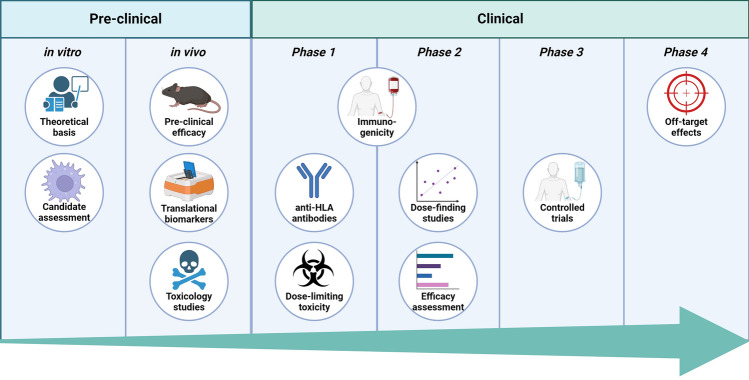
Key domains by which the performance of AAMs for the treatment in ALI will be assessed are focussed on safety and efficacy. For safety: there must be no clinical concern for dose-limiting toxicity, the impact of any anti-HLA antibody formation on transplant suitability, immunogenicity (e.g. macrophage activation syndrome, transfusion-associated graft versus host disease), or off-target effects on other tissues. For efficacy: the mechanism of action must have robust pre-clinical studies to support the claims, biomarker studies will be required (and may require historic controls, or novel methods of interpretation). *Pharmacokinetic studies and tissue biopsy may not be achievable in clinical trials of patients with ALI for clinical and ethical reasons. Image created with biorender.com

As Fig. 2 shows, there are multiple criteria by which AAMs will be judged prior to widespread adoption in humans as a treatment for ALI.

### Immunogenicity

Allogeneic cell products (i.e. blood transfusions) are administered to patients every day in hospitals. However, the potential for allogeneic AAMs to exhibit greater immunogenicity, or cause an off-target effect such as transfusion-related lung injury cannot be discounted. There remains a theoretical risk of transfusion-associated graft versus host disease (TAGvHD), due to complete depletion of donor T cells being impossible, though there is extensive data to support the cell numbers being much lower than typically associated with TAGvHD, and AAMs have been shown to attenuate the inflammatory processes in GvHD animal models (Hutchinson et al. 2014; Hanaki et al. 2021). Whilst this can be mitigated against by ensuring donor/recipient HLA mismatch for phase 1 trials, in which there is a duty of care to minimise risk as far as possible, it is unclear if regulators will require this level of product matching in later phase trials, as it would represent a burden of proof to which other blood products containing the same quantities of T lymphocytes are not subjected. The unscheduled workload for histocompatibility and immunogenetics laboratories, therefore, has the potential to be significant.

Patients with ALI have the potential to progress to ALF and be considered for OLT. There is a risk that an allogeneic product may lead to the development of novel anti-HLA antibodies which reduces the pool of suitable donor livers. Clinical trials of allogeneic products will provide evidence regarding the likelihood of this, but until a dosing level of AAMs is established, the risk cannot be accurately quantified (Tran et al. 2023). The development of persistent HLA class 2 antibodies has been demonstrated in the infusion of other allogeneic cell products (regulatory dendritic cells) (Tran et al. 2023). However, in this study, there were no differences in acute liver rejection at 12 months, and the relative importance of donor-recipient HLA matching on clinical outcomes for OLT remains debated (Thomson et al. 2020).

Macrophage activation syndrome (MAS) is a theoretical concern if the polarisation of AAMs changes to pro-inflammatory. The criteria for MAS require a fever, and include raised ferritin, low platelets, raised aspartate aminotransferase, raised triglycerides and low fibrinogen (Ravelli et al. 2016). Whilst MAS has never been reported in macrophage trials to date, all of these diagnostic criteria have been reported in APAP ALI, posing a diagnostic challenge. With time, we expect that this risk will be shown to be entirely theoretical in the context of AAMs given their pro-resolution phenotype.

### Managing complications

The delivery of a new class of medications for a condition as common as APAP ALI will require clinician education, and post-market surveillance to ensure that adverse effects are identified and managed correctly. Mechanism-specific theoretical concerns for cell therapies include cell aggregation causing pulmonary embolism and transfusion-related acute lung injury, but the standard potential hazards of blood product transfusion will still apply (Hutchinson et al. 2014).

## Conclusion

The pre-clinical evidence presented here provides the rationale for testing allogeneic AAMs as a therapeutic product for APAP ALI in humans. There is a clear unmet need, a sound pre-clinical basis supporting clinical trials, existing trial data suggesting autologous macrophage therapy is safe and effective, and ongoing recruitment which hopes to demonstrate product safety, and allow exploration of efficacy. If this proof-of-concept regenerative therapeutic is demonstrated to be safe in humans with APAP ALI, it could conceivably be tested in many other pathologies characterised by inflammation and necrosis.

There are risks with potential to impact the success of AAMs, but the field of regenerative medicine and regulatory frameworks are now sufficiently well developed to allow these to be anticipated and addressed.

By articulating what the requirements of AAMs for ALI will be; an allogeneic, ex vivo polarised AAM treatment, which can be produced (and is tolerable to ALI patients) at a dose needed to be clinically effective—the remaining knowledge gaps can be identified. It is our hope that this article has described how those gaps will be addressed, and that the science to date suggests that studies may well identify a new therapeutic option for the treatment of ALI.
